# Phase I Dose-Escalation Study of Once Weekly or Once Every Two Weeks Administration of High-Dose Sunitinib in Patients With Refractory Solid Tumors

**DOI:** 10.1200/JCO.18.00725

**Published:** 2018-12-26

**Authors:** Maria Rovithi, Sophie L. Gerritse, Richard J. Honeywell, Albert J. ten Tije, Rita Ruijter, Godefridus J. Peters, Jens Voortman, Mariette Labots, Henk M.W. Verheul

**Affiliations:** ^1^Vrije Universiteit Medical Center, Amsterdam, the Netherlands.

## Abstract

**PURPOSE:**

Dose and schedule optimization of treatment with tyrosine kinase inhibitors is of utmost importance. On the basis of preclinical data, a phase I clinical trial of once weekly or once every 2 weeks administration of high-dose sunitinib in patients with refractory solid malignancies was conducted.

**PATIENTS AND METHODS:**

Patients with advanced cancer refractory to standard treatment were eligible. With use of a standard 3 + 3 phase I design, patients received escalating doses of sunitinib, in 100 mg increments, starting at 200 mg once weekly. In both the once weekly and once every 2 weeks cohorts, 10 more patients were included at the maximum tolerated dose level. Primary end points were safety and tolerability.

**RESULTS:**

Sixty-nine patients with advanced cancer, predominantly colorectal cancer (42%), were treated with this alternative dosing regimen. Maximum tolerated dose was established at 300 mg once weekly and 700 mg once every 2 weeks, resulting in nine- and 18-fold higher maximum plasma concentrations compared with standard dose, respectively. Treatment was well tolerated, with fatigue (81%), nausea (48%), and anorexia (33%) being the most frequent adverse events. The only grade 3 or 4 treatment-related adverse event in 5% or more of patients was fatigue (6%). Sixty-three percent of patients had significant clinical benefit, with a 30% progression-free survival of 5 months or more.

**CONCLUSION:**

Sunitinib administered once weekly at 300 mg or once every 2 weeks at 700 mg is feasible, with comparable tolerability as daily administration. Administration of 700 mg once every 2 weeks can be considered as the most optimal schedule because of the highest maximum plasma concentration being reached. The promising preliminary antitumor activity of this alternative schedule in heavily pretreated patients warrants further clinical evaluation and might ultimately indicate a class characteristic of tyrosine kinase inhibitors.

## INTRODUCTION

Treatment with tyrosine kinase inhibitors (TKIs) often results in durable clinical responses and survival benefit with an acceptable safety profile for patients with advanced malignancies.^1^ Nonetheless, resistance to TKIs eventually develops in all patients. The current use of TKIs is based on the principle that prolonged drug exposure is necessary for optimal antitumor activity because of continuous inhibition of angiogenesis and specific intracellular signaling.^2^ However, the occurrence of toxicity leads to a narrow therapeutic window, which impedes additional dose escalation and drug exposure.^3^ It is hypothesized that the clinical efficacy of these agents might be further improved by optimization of treatment schedules and dosing.^4^ Disease progression has been attributed to subtherapeutic levels, whereas dose escalation might overcome the initial development of resistance,^5^ and increased exposure correlates with improvement in clinical benefit.^6^

Sunitinib malate (SUTENT; Pfizer, New York, NY) is an orally administered TKI that targets multiple kinase receptors, including the vascular endothelial growth factor (VEGF) receptor and platelet-derived growth factor receptor. It is approved for patients with renal cell cancer, GI stromal cell tumors (GISTs), or pancreatic neuroendocrine tumors in a dose of 50 mg per day for 4 weeks followed by a 2-week off period or a continuous regimen of 37.5 mg doses per day.^7^

Clinical feasibility and safety of higher sunitinib doses were previously reported when a single dose of sunitinib up to 300 mg was safely administered to patients.^8^ We hypothesized that intermittent, high-dose administration of sunitinib might ultimately result in higher plasma and subsequent intratumoral concentrations, leading to enhanced efficacy. We have previously demonstrated that short exposure to high concentrations of sunitinib leads to complete inhibition of tumor cell proliferation in vitro and significantly impairs tumor growth in vivo compared with continuous lower exposure.^9^ Other scientific support for the role of TKI dose escalation comes from a meta-analysis that indicated the proportional relationship between drug exposure and the probability of response.^6^ Recently, intrapatient dose escalation at the time of progression in patients who were receiving sunitinib was shown to overcome resistance, although transiently, and resulted in an increase in progression-free survival of greater than or equal to 5 months.^5^

Identification of optimal treatment strategies is actively pursued for other TKIs. Comparable to our study with sunitinib, weekly 10-fold–higher doses of erlotinib have been reported as salvage therapy in patients with non–small-cell lung cancer and leptomeningeal metastases with an acceptable toxicity profile.^10^ Imatinib dose escalation has been suggested as an effective therapy for advanced GIST after progression on standard dose in patients who harbor exon 9 mutations, which underscores the need for dose individualization.^11^ Almost all patients with imatinib-resistant metastatic GIST eventually develop resistance to treatment with sunitinib, generally within 1 year, which results in disease progression.^12^ Mechanisms of GIST resistance to sunitinib treatment are largely unknown.^13^ Because sunitinib targets a broader spectrum of kinases compared with imatinib, additional mechanisms possibly play a role in the acquisition of resistance. The promising antitumor activity of this high-dose sunitinib strategy potentially could overcome therapy resistance to sunitinib in these patients similar to those observed with imatinib.^14^ On the basis of these preclinical and clinical findings, the current phase I clinical trial was conducted to investigate the maximum tolerated dose (MTD) of once weekly or once every 2 weeks administration of sunitinib, the safety and clinical feasibility, the pharmacokinetic parameters, and the preliminary efficacy in patients with advanced solid malignancies refractory to standard treatment.

## PATIENTS AND METHODS

### Patient Eligibility

Eligible patients included adults with histologically confirmed advanced solid tumors that were progressive after standard treatment. Major inclusion and exclusion criteria are listed in the Data Supplement. The study was conducted in accordance with the Declaration of Helsinki and Good Clinical Practice guidelines. Before inclusion, patients provided informed consent on the study protocol approved by the local institutional review board.

### Study Design and Treatment Plan

This dose escalation, phase I, single-institution clinical trial was conducted at the Vrije Universiteit Medical Center, Amsterdam, the Netherlands. A standard 3 + 3 design was used with a starting dose cohort of 200 mg sunitinib administered orally once weekly and escalating in increments of 100 mg. Patients continued sunitinib until progression, intolerance, or consent withdrawal. The primary objective was to determine the MTD and evaluate the safety and tolerability, whereas secondary objectives were to assess the pharmacokinetic parameters of this scheduling and preliminary assessment of the efficacy of sunitinib intermittent treatment. Once weekly MTD level was subsequently set as the starting dose level for the once every 2 weeks schedule, which followed the same design principles. Patients were considered evaluable (for toxicity, pharmacokinetics, and response) who completed a minimum of 2 weeks of sunitinib treatment, which means two administrations of sunitinib on the once weekly schedule and one administration of sunitinib in the once every 2 weeks schedule, including 2 weeks of follow-up. After determination of the MTD, both schedules expanded to include 10 additional patients at the MTD level to evaluate preliminary efficacy.

### Safety Assessment

Physical condition assessments, including ECG and blood hematology and chemistry, were performed weekly during the first 8 weeks and once every 4 weeks thereafter. Adverse events (AEs) were monitored throughout the study and graded according to the National Cancer Institute Common Terminology Criteria for Adverse Events (version 4.0). MTD was defined as the highest dose level at which less than or equal to 33% of patients experienced dose-limiting toxicities (DLTs). DLT was defined as any grade 3 or higher toxicity attributable to sunitinib that occurred during the first 6 weeks of therapy.

### Pharmacokinetics

Blood samples for pharmacokinetic assessments were collected pretreatment and subsequently at multiple time points (0, 2, 4, 6, 8, 10, and 24 hours postdose on day 1 for both time schedules and thereafter at days 3, 8, 10, 15, 17, and 22 for the once weekly schedule and at days 3, 15, 17, and 29 for the once every 2 weeks schedule). Sunitinib plasma concentrations were determined by liquid chromatography-mass spectrometry.^15^ To evaluate sunitinib exposure, peak concentration, half-life, area under the concentration-time curve, and time to half-life were calculated with the validated PKSolver add-in for Microsoft Excel 2010 (Microsoft Corporation, Redmond, WA).

### Treatment Efficacy

All patients underwent computed tomography scanning at baseline and subsequently every 8 weeks for evaluation of efficacy. Antitumor response was evaluated by Response Evaluation Criteria in Solid Tumors (RECIST) version 1.1. PFS was defined as the time from the date of the first dose of study medication to the date of first disease progression or the date of death.

### Statistical Analysis

Descriptive statistics were used for baseline characteristics, safety assessment, and pharmacokinetic data. Data are expressed as mean ± standard deviation when appropriate.

## RESULTS

### Patients

A total of 73 patients had been included in the study of whom 71 started study medication and 69 received sunitinib for 2 weeks or more. The baseline patient characteristics are listed in Table 1. Median age was 62 years (range, 29 to 85 years), and 64 patients (88%) had a WHO performance status of 1. Colorectal cancer (CRC) was the most frequent tumor type (42%). All patients were pretreated extensively, with 67% having received two or more previous treatment lines. The reason for not starting study medication for two patients after enrollment was rapid deterioration in clinical status. In the escalation cohorts, 12 patients at the MTD (six in the 300 mg once weekly cohort and six in the 700 mg once every 2 weeks cohort) received (at least) every dose that was scheduled in the first 6 weeks of therapy (DLT window). In the expansion cohort, another 20 patients (10 in each cohort) received at least 6 weeks of sunitinib treatment.

**TABLE 1.**
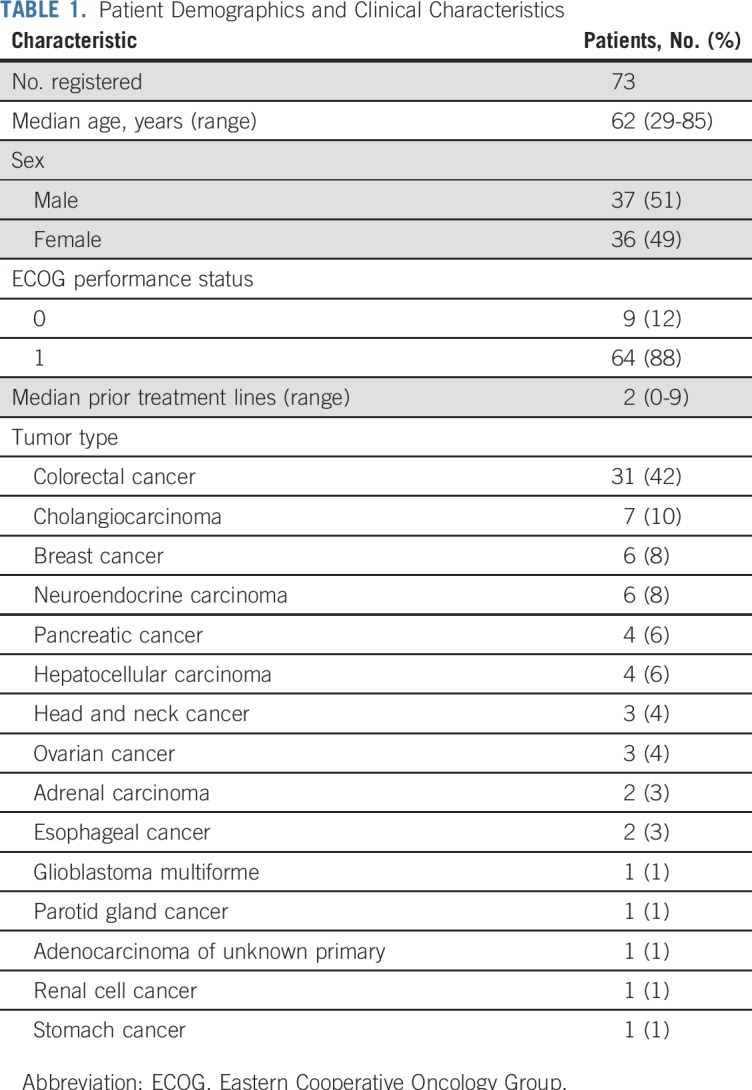
Patient Demographics and Clinical Characteristics

### Safety

High-dose, intermittent sunitinib demonstrated a toxicity profile comparable to the standard flat dose. Table 2 lists all clinically significant toxicities that occurred in 10% or more of the 69 patients evaluable for toxicity (grade 1 or 2) and all grade 3 or 4 treatment-related AEs. The most commonly observed AEs of any grade were fatigue (n = 56; 81%), nausea (n = 33; 81%), and anorexia (n = 23; 33%). With regard to grade 3 or higher AEs, four patients (6%) experienced grade 3 fatigue. The majority of adverse effects were constitutional and manageable with standard supportive care interventions. Severe bowel toxicity (presacral abscess) was observed in one patient who received the first dose level (200 mg) in the once weekly cohort (outside the DLT period), whereas one patient who received the third dose level (400 mg once weekly) presented with a fatal bowel perforation (during the DLT period). Both patients had been irradiated previously in the affected area. These serious AEs led to an amendment of the study protocol to exclude patients with a history of pelvic, thoracic, and extended vertebral irradiation. After the amendment, at the dose level of 400 mg once weekly, DLTs occurred in two patients: One developed grade 3 bile stasis, and one developed grade 3 fatigue. One patient on the 400 mg once weekly schedule and with CRC and extensive peritoneal carcinomatosis with a substantial clinical benefit lasting longer than 7 months developed a bowel perforation at the time of disease progression as a result of a growing peritoneal tumor lesion. He underwent a successful operation. The dose level of 300 mg weekly was expanded to six patients and no additional DLTs were observed. Therefore, the MTD was determined at 300 mg once weekly.

**TABLE 2.**
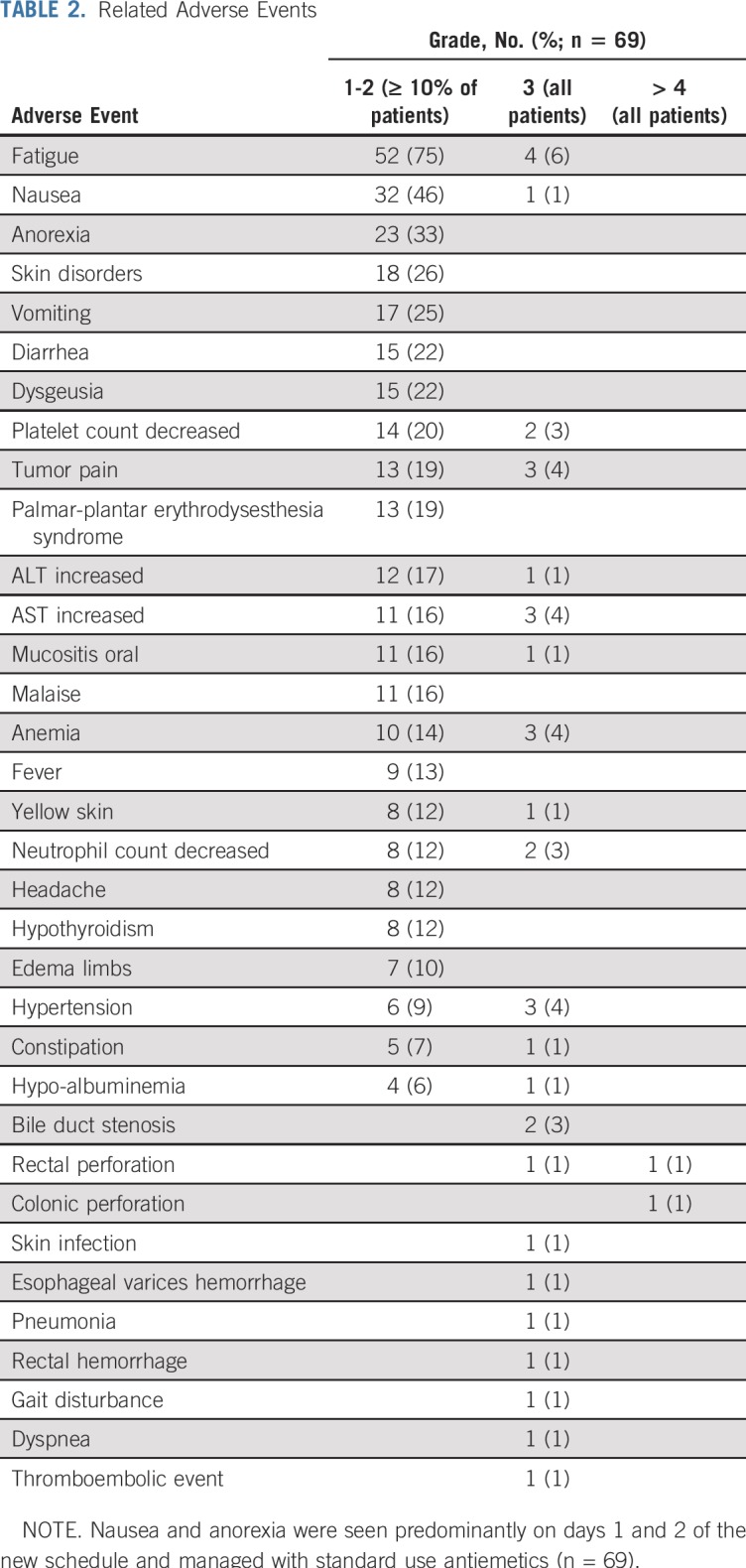
Related Adverse Events

Subsequently, enrollment in the once every 2 weeks schedule was initiated at the MTD level of the once weekly schedule, that is, 300 mg once every 2 weeks, and escalated in steps of 100 mg. Because patients experienced no DLTs, doses were escalated up to 800 mg once every 2 weeks in subsequent cohorts. In the 800-mg cohort, two patients experienced a DLT: One developed a combination of a grade 3 increase in ALT and AST, and one developed a grade 3 hepatobiliary disorder (bile duct obstruction). The MTD of the administration of sunitinib once every 2 weeks was set at 700 mg. Dose escalation steps and DLTs are listed in Appendix Table A1 (online only).

### Pharmacokinetics

**FIG 1.**
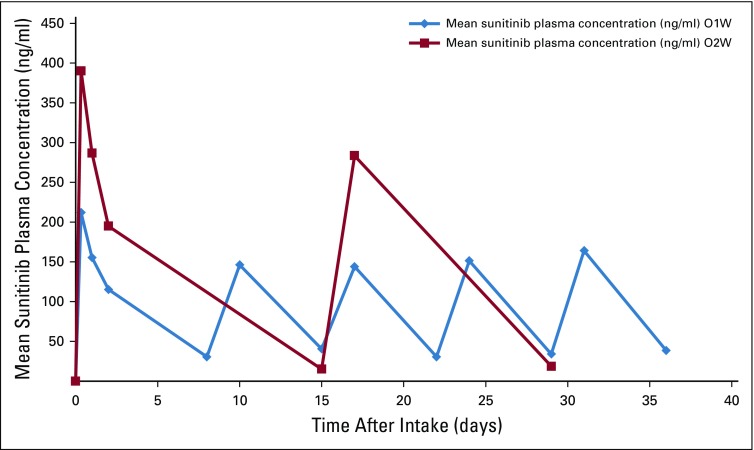
Mean sunitinib plasma concentrations of all evaluable patients after intake of the drug once per week and once every 2 weeks. Mean sunitinib plasma concentrations of all evaluable patients after intake of the drug once per week (O1W) and once every 2 weeks (O2W).

Pharmacokinetic parameters of sunitinib^16^ are listed and compared with standard scheduling in Table 3. Exposure to sunitinib, as designated by an increase in the maximum plasma concentration (C_max_), increased across dose levels (0.167 ± 0.06, 0.261 ± 0.12, and 0.219 ± 0.07 μg/mL for the 200-, 300-, and 400-mg dose levels once weekly, respectively, and 0.215 ± 0.12, 0.400 ± 0.12, 0.301 ± 0.16, 0.357 ± 0.12, 0.505 ± 0.15, and 0.551 ± 0.28 μg/mL for the 300, 400, 500, 600, 700, and 800 mg dose levels once every 2 weeks, respectively). C_max_ was achieved in approximately 2 to 8 hours and provided nine to 18 times higher peak concentrations than standard dosing.^16^ Although significant interpatient variability was noted, intrapatient variability was minimal, and almost no plasma drug accumulation of sunitinib occurred in time. The mean terminal half-life of sunitinib was approximately 44 hours. The majority of patients (97%) reached a C_max_ higher than 0.1 μg/mL (250 nM). Dose escalation led to a proportionate increase in drug exposure (Fig 1). Pharmacokinetics of the metabolite of sunitinib (SU12662) followed the same pattern of sunitinib itself and are both listed in Appendix Table A2 (online only).

**TABLE 3.**
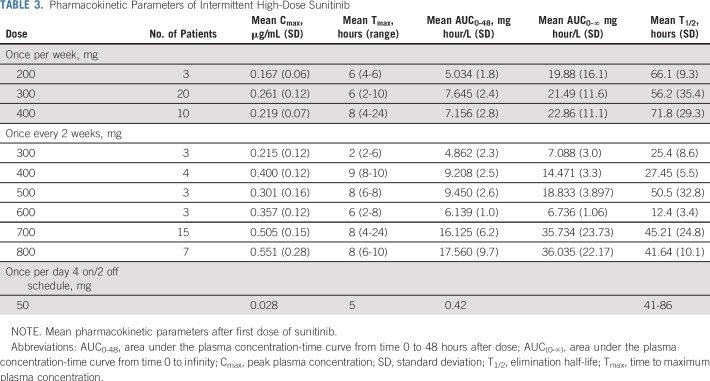
Pharmacokinetic Parameters of Intermittent High-Dose Sunitinib

### Treatment Efficacy

**FIG 2.**
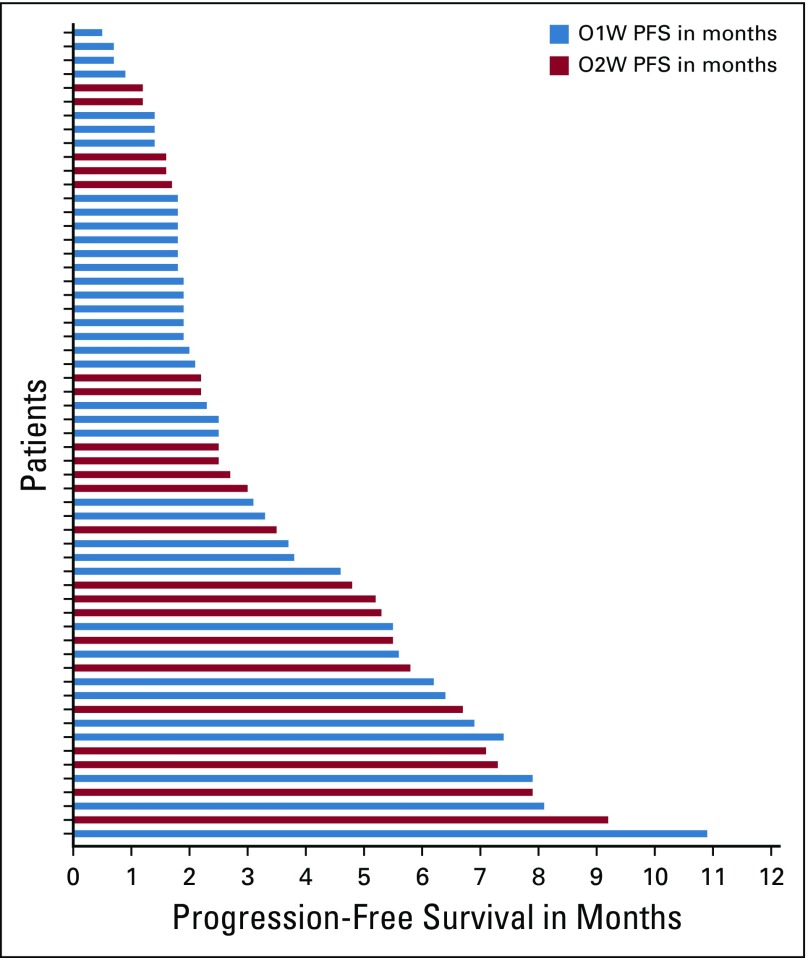
Progression-free survival (PFS) for all evaluable patients after once per week and once every 2 weeks administration of sunitinib.

Clinical benefit from treatment was characterized by prolonged disease stabilization, tumor marker response, and improvement of disease-related symptoms. Of 69 patients, 59 were evaluable for response. Of the other 10 patients, five presented with a DLT, two withdrew consent at week 3 for nonmedical reasons, two stopped because of a non–treatment-related serious AE, and treatment of one patient was discontinued by the investigator when the study was put on hold to await for the amendment. Thirty-seven patients (63%) had clinical benefit defined as progression free at 2 months of treatment, whereas 30% (18 of 59) had stable disease for 5 months or longer (Fig 2). Mean PFS in the MTD group was 3.5 months (range, 0.5 to 9.2 months). The range for the duration of treatment (all patients) was 0.5 to 47 weeks. The median duration of therapy was 8 weeks, and the mean was 14 weeks.

**FIG 3.**
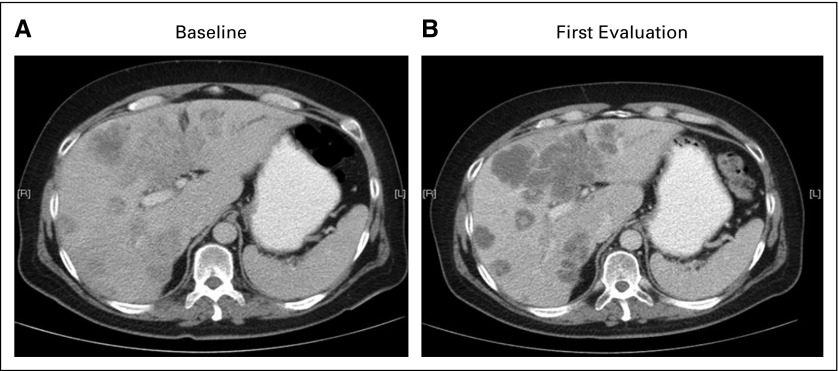
Stable disease in a patient with metastatic colorectal cancer during treatment. Computed tomography scans (A) before treatment and (B) at first evaluation (8 weeks). The on-treatment scan indicates tumor necrosis characterized by homogeneous hypo-attenuation and sharp tumor-liver interface (Data Supplement).

Despite RECIST version 1.1 stable disease on computed tomography evaluation, on-treatment scans were indicative of tumor necrosis in most patients with clinical benefit characterized by homogenous hypo-attenuation and sharp tumor-liver interface^17^ (Fig 3). When response evaluation was based on modified Choi criteria,^18^ a 32% response rate was reached for the 59 patients at the time of first evaluation.

Twenty-seven of all participating patients had detectable blood tumor markers (eg, carcinoembryonic antigen, CA15.3, CA19.9 [greater than the upper limit of normal]). Compared with baseline, the eight patients with the longest time on treatment (5 months or more PFS) had a significant tumor marker decrease at first evaluation compared with baseline (−35% ± 22%; *P* = .02). No tumor marker response was seen in the 10 patients who progressed (an increase of 94 ± 124%; *P* = .2). Almost all patients (96%) presented with a significant tumor marker increase 24 to 72 hours after first dose ingestion.

## DISCUSSION

Treatment strategies with multitargeted TKIs have been focused on continuous drug exposure at their MTD for optimal target inhibition.^3^ This continuous inhibition of targeted signaling pathways has been proposed as key for their mechanism of action leading to clinical antitumor activity.^19^

Proof of concept for this dogma is lacking mainly because of the difficulty to measure adequately true inhibition of specific drug targets in patient tumor samples. Dissimilarly, conventional cytotoxic approaches include the administration of intense, intermittent doses.^20^ In this study, we aimed to reach the highest tolerable peak plasma concentrations with the subsequent highest intratumoral peak concentrations for the drug to exert direct cytocidal effects and suffice to block targets with a lower drug-binding affinity at the tumor level on the basis of preclinical experiments.^9^ On the basis of this hypothesis, we conclude that administration of 700 mg of sunitinib once every 2 weeks, the dose at which the highest C_max_ is received, is the optimal alternative high-dose treatment schedule.

The sunitinib daily dosing strategy was established in the original first-in-human, phase I study where responses were reported in five patients.^16^ Phase III studies of sunitinib standard dosing, with 4 weeks 50 mg per day administration followed by 2 weeks treatment interruption, resulted in the approval of sunitinib to treat patients with metastatic renal cell cancer, imatinib-resistant GIST, and advanced neuroendocrine pancreatic cancers with significant clinical and overall survival benefit.

In the current phase I clinical trial, sunitinib administration at a dose of 300 mg once weekly or 700 mg once every 2 weeks was shown to be safe and tolerable with a comparable toxicity profile as the standard sunitinib schedule of 50 mg given for 28 days with a 14-day break recently reported in the Alliance 031203 CABOSUN trial.^21^ In our phase I trial, grade 3/4 and 5 AEs related to intermittent high-dose sunitinib (once weekly or once every 2 weeks) occurred in 46% and 2% of patients compared with 68% and 7% of patients treated with the standard sunitinib schedule in the CABOSUN trial. The most frequently reported grade 3/4 toxicity was fatigue, which occurred in four (6%) of 69 patients in our phase I trial compared with 11 (15.3%) of 72 patients in the CABOSUN trial. The AEs of nausea and anorexia were predominantly seen on days 1 and 2 of the new schedule and well manageable with standard use of antiemetics.

Pharmacokinetic evaluation in our trial clearly indicated that intermittent high peak concentrations could be reached without detrimental toxicity because the majority of the patients reached a C_max_ greater than 0.1 μg/mL (250 nM). Patients in the standard scheduling with trough concentrations of more than 0.1 μg/mL exhibited DLTs.^22^ We consider these peak concentrations as most likely responsible for the direct antitumor activity in accordance with preclinical findings.^9^

Administration of this alternative scheduling was complicated by the development of serious bowel toxicity in two patients who both received prior radiotherapy. Radiotherapy has previously been reported as a contributing factor to serious bowel toxicity seen with concomitant anti-angiogenic treatment, including sunitinib specifically.^23,24^ The underlying mechanisms are still to be elucidated. The working hypothesis assumes inadequate capability of tissue repair after radiation-induced bowel injury because of an impaired VEGF response.^25^ After exclusion of patients who were previously irradiated at the bowel region, no additional serious bowel toxicity was observed.

Recently, Chien et al^26^ translated data from mouse models in which intermittent high- dose lapatinib resulted in improved efficacy compared with the standard continuous low- dose therapy. These investigators reported a phase I trial that investigated this schedule in patients with advanced solid tumors. High-dose, intermittent lapatinib was well tolerated and resulted in significantly increased plasma concentrations. In addition, a relationship between lapatinib exposure and biologic activity was established; patients with plasma concentrations approximating 10 μg/mL presented with marked responses, whereas all patients with low lapatinib plasma concentrations had progressive disease.^26^

In this trial, 20% of the evaluable patients with CRC reached a PFS of more than 5 months (range, 5.5 to 12.5 months), which is notable because in the original phase I study, none of the three patients with CRC showed a long-lasting response or tumor stabilization.^16^ In a subsequent phase II clinical trial, sunitinib was evaluated in the metastatic CRC setting where 16% of patients with CRC reached a PFS of 5 months or more^27^ and concluded that daily sunitinib monotherapy provides no significant clinical benefit; therefore, further development of sunitinib for CRC was terminated. Although the only approved TKI for CRC, regorafenib shows a small, but significant overall survival benefit with a challenging toxicity profile.^28^ Our results indicate that alternative dosing of sunitinib may provide a promising strategy for the treatment of patients with advanced CRC with an acceptable toxicity profile.

To determine the efficacy of pulsatile, high-dose sunitinib in patients with metastatic CRC, we will start shortly a prospective, randomized, open-label, phase II/III clinical trial to compare 700 mg sunitinib once every 2 weeks with standard treatment with Trifluridine plus tipiracil (TAS-102) in patients with metastatic adenocarcinoma of the colon or rectum who are refractory or intolerant to therapy with fluorouracil, oxaliplatin, and irinotecan anti-VEGF therapy (and anti–epidermal growth factor receptor therapy in KRAS wild type).

In conclusion, pulsatile, high-dose sunitinib was well tolerated and exhibited preliminary clinical significant benefit in patients with refractory solid malignancies, which warrants its development. The once every 2 weeks administration of 700 mg sunitinib is feasible and the most optimal schedule because of the highest C_max_ being reached. Our study highlights the importance to improve further the efficacy of this new class of agents by optimization of dose and scheduling strategies.

## Figures and Tables

**TABLE A1.**
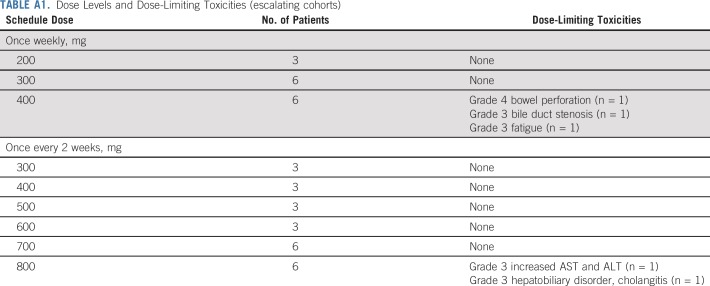
Dose Levels and Dose-Limiting Toxicities (escalating cohorts)

**TABLE A2.**
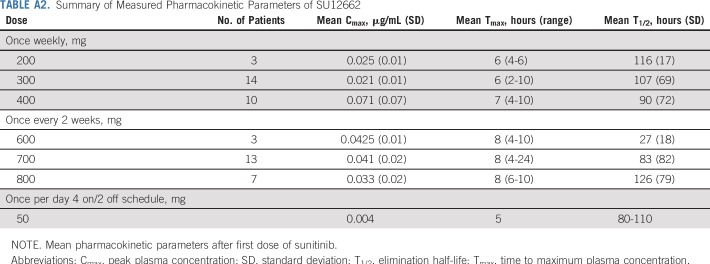
Summary of Measured Pharmacokinetic Parameters of SU12662
